# Optimization of Ozonation Process for the Reduction of Excess Sludge Production from Activated Sludge Process of Sago Industry Wastewater Using Central Composite Design

**DOI:** 10.1100/2012/239271

**Published:** 2012-04-19

**Authors:** B. Subha, M. Muthukumar

**Affiliations:** Environmental Engineering and Technology Laboratory, Department of Environmental Sciences, Bharathiar University, Coimbatore 641046, India

## Abstract

Sago industries effluent containing large amounts of organic content produced excess sludge which is a serious problem in wastewater treatment. In this study ozonation has been employed for the reduction of excess sludge production in activated sludge process. Central composite design is used to study the effect of ozone treatment for the reduction of excess sludge production in sago effluent and to optimise the variables such as pH, ozonation time, and retention time. ANOVA showed that the coefficient determination value (*R* ^2^) of VSS and COD reduction were 0.9689 and 0.8838, respectively. VSS reduction (81%) was achieved at acidic pH 6.9, 12 minutes ozonation, and retention time of 10 days. COD reduction (87%) was achieved at acidic pH 6.7, 8 minutes of ozonation time, and retention time of 6 days. Low ozonation time and high retention time influence maximum sludge reduction, whereas low ozonation time with low retention time was effective for COD reduction.

## 1. Introduction

Sago, the edible starch globules processed from the tubers of tapioca (*Manihot esculenta*), is the staple diet of middle income populations in India. There are 3,226 industries in Tamil Nadu, of these 1,522 are small, 388 medium, and 205 are of larger scale. They produce about 15 to 30 tonne of sago per unit/day and discharge about 40,000 to 50,000 litres of sago wastewater per tonne of sago [1]. Sago manufacturing industrial units, both at medium and large scale, suffer from inadequate treatment and disposal problems. They generate more than 85% of the total wastewater output, and about 400 units discharge directly into rivers. Sago wastewater is complex and acidic in nature with high organic matter, intense COD and BOD, suspended solids, obnoxious odour, and irritating colour [2].

 Some widely used methods to treat sago wastewater are high-rate anaerobic treatment such as anaerobic filters and fluidized beds [1], hybrid upflow anaerobic sludge blanket (HUASB) reactor [3], anaerobic tapered fluidized bed reactor [4], hybrid reactor [5], and three-phase fluidized bed bioreactor [6]. Other biomanagement methods adopted to treat the sago wastewater are using fungi (*G. putredinis, Trichoderma harzianum*) and bacteria (*Alcaligenes, Bacillus, and Corynebacterium*) [2, 7].

Ozonation is an alternative process for treating the wastewater. Since ozone is a very powerful oxidant (2.07 V for ozone *versus *2.8 V for hydroxyl radical) mainly used for disinfection process, it has a strong cell lytic activity that can kill the microorganisms found in the wastewater [8, 9]. Once dissolved in water, ozone reacts with large number of organic compounds in two possible ways: direct oxidation, as molecular ozone, or indirect reaction through the formation of secondary oxidants such as free radicals, particularly hydroxyl radical.

Several methods such as promoting cryptic growth, ultrasounds, heat, alkali, and ozone treatment have been developed for wastewater sludge treatment [10, 11]. Among these, ozonation was referred as one of the most cost-effective technologies [12, 13] used for domestic wastewater [14], cork-processing wastewater [15], and coke-oven wastewater [16].

The statistical tool of response surface methodology (RSM) has been proposed to include the influence of individual factors as well as their interactive effects. It is employed for multiple regression analysis using quantitative data obtained from properly designed experiments to solve multivariable equations. A further benefit of using the RSM is the reduction of the number of experiments needed to compare a full experimental design at the same level [17]. RSM has been successfully used in central composite design to model and optimize ozonation process [18]. Though previous studies have been carried out for sludge reduction using various treatment methods, sago industrial wastewater has not been examined previously for ozonation process. Hence, the objective of this paper is to study the reduction of excess sludge production in activated sludge process of sago effluent employing ozonation process and optimizing the process variables such as pH, ozonation time, and retention time on sludge reduction using RSM.

## 2. Materials and Methods

### 2.1. Effluent Source

The raw effluent was obtained from a conventionally activated sludge system in SPAC Tapioca treatment plant in Erode district, Tamil Nadu, India. Sample collection and characterization was performed according to the standard methods [19], and the initial parameters analyzed are given in Table 1.

### 2.2. Experimental Setup

The experimental setup consisted of an oxygen concentrator (Sim O_2_ plus, Italy), ozone generator (Ozonetek Ltd., India) with built-in oil-free compressor and reaction column. A controlled flow rate of 2 l/min of oxygen was used to produce 2 g/h of ozone. The reactor had a glass column of 72 cm height, outer diameter of 4.5 cm, and an inner diameter of 3.5 cm and having a capacity to hold 1500 mL of effluent. It was provided with a sample port at various points, an ozone gas inlet at the bottom with an air diffuser over the inlet port to diffuse the oxygen/ozone gas mixture through the column, and a closed top with a collection port to collect the unreacted ozone gas venting it out. Teflon tube was used for connecting the ozone outlet port from the ozone generator to the ozone reaction chamber.

### 2.3. Experimental Procedure

 The collected effluent was transferred into the laboratory-scale batch reactor (20 L capacity) at room temperature. The samples were then aerated using an aerator (Shengze, BS 410, China) at a flow rate of 1 litre per minute (LPM) which was controlled by a Rota meter (Orient Hardware, Coimbatore, India). Mechanical stirrer was used at a speed of 100 rpm to ensure complete mixing of the influent. Biosludge was continuously fed to the aeration basin by a peristaltic pump (Enter Tech, Mumbai, India) to maintain the biomass concentration. Once the biomass concentration attained a value of 2000 mg L^−1^, the sample was transferred into 500 mL conical flask, and pH was adjusted according to the design of experiment using 1 N HCl and 1 N NaOH using a pH meter (Susima Technologies, AP 1 plus, Chennai, India). The sample was then transferred to the ozonation chamber, and 66.6 mg O_3_/min was passed into the chamber. The ozonation time varied from 1 to 20 minutes, and after ozonation it was transferred in to 500 mL conical flask sealed tightly with a rubber cork. The experiment was carried out at room temperature, and the retention time was 10 days. 

### 2.4. Experimental Design-Central Composite Design (CCD)

Response surface methodology (RSM) is a collection of statistical tools and techniques for exploring an approximate functional relationship between a response variable and a set of design variables [20]. A three-level factorial design was established with the help of the Design Expert software (Central Composite Design Expert Version 8.0.3, Stat Ease, Minneapolis, USA). In the experimental design, model parameters were estimated by forming an optimal plan matrix using a second-degree quadratic polynomial equation:

(1) $$\begin{array}{cc} Y & {=B}_{o}+B_{1}X_{1}+B_{2}X_{2}+B_{3}X_{3}{+\;B}_{11}X_{1}^{2}\\ & \;+B_{22}X_{2}^{2}+B_{33}X_{3}^{2}+B_{12}X_{1}X_{2}\\ & \;+B_{13}X_{1}X_{3}+B_{23}X_{2}X_{3}, \end{array}\;\;$$

where *Y* is predicted response, *B*_*o*_ the constant coefficient, *B*_1_, *B*_2_, and *B*_3_ the linear coefficient, *B*_11_, *B*_22_, *B*_33_ the quadratic coefficient, *B*_11_, *B*_12_, *B*_13_ the cross-products coefficient, and *X*_1_, *X*_2_, and *X*_3_ were input variables (pH, ozonation time and retention time). The variables and their levels are designated as −1.682, −1, 0, +1, and +1.682 given in Table 2. According to (1), it was found that a total of 20 runs are necessary to optimize the response. Adequacy of the proposed model was then revealed using the diagnostic checking tests provided by analysis of variance (ANOVA). The quality of the fit polynomial model was expressed by the coefficient of determination *R* ^2^, adjusted *R* ^2^, and “adequate precision.” The fitted polynomial equation was expressed as three-dimensional (3D) surface plots to visualize individual and interactive effect of factors on the response within the design range. The optimum region was also identified based on the main parameters in the overlay plot [21]. 

### 2.5. Analytical Procedure

Volatile suspended solids (VSSs) and chemical oxygen demand (COD) were measured in accordance with standard methods [19]. The biomass settling characteristics were determined based on the sludge volume (30 min settling period). The Total suspended solids (TSSs) and mixed liquor suspended solids (MLSSs) were determined by drying the sample at 105°C. Phosphate, nitrate, and sulphate were analyzed using spectrophotometer (Hitachi, model U-3210, Tokyo) according to Saxena [22]. Carbohydrate and starch were determined by anthrone method [23]. VSSs and COD were analysed periodically based on the design of experiment as shown in Table 3. 

## 3. Results and Discussion 

### 3.1. Statistical Analysis and Fitting of Second-Order Polynomial Equation

Response surface methodology is an empirical modelling technique, which is used to evaluate the relationship between a set of controllable experimental factors and observed results [24]. Several factors influence the sludge reduction from sago effluents of which pH, ozonation time, and retention time play a vital role. In order to study the effect of these variables, central composite design was used. The regression equations given below (2) and (3) are obtained by the analysis of variance giving the percentage level of VSS and COD reduction:

(2) $$\begin{array}{cc} & \text{\%}\;\text{VSS}\;\text{reduction}\;\left(Y_{1}\right)\\ & \;=76.31-2.67X_{1}+4.56X_{2}+21.12X_{3}+1.50X_{1}^{2}\\ & \;\;-0.35X_{2}^{2}-5.12X_{3}^{2}+1.22X_{1}X_{2}+2.76X_{1}X_{3}\\ & \;\;+0.59X_{2}X_{3}, \end{array}$$

(3) $$\begin{array}{cc} & \text{\%}\;\text{COD}\;\text{reduction}\;\left(Y_{2}\right)\\ & \;=86.17-0.71X_{1}-1.13X_{2}+3.16X_{3}-3.63X_{1}^{2}\\ & \;\;-3.07X_{2}^{2}-8.78X_{3}^{2}-0.91X_{1}X_{2}-1.54X_{1}X_{3}\\ & \;\;-6.99X_{2}X_{3}. \end{array}$$

In the two models of *Y*_1_ and *Y*_2_, the probability value of <0.0001 and 0.0013 implies that these models were significant. The ANOVA results for the parameters *Y*_1_ and *Y*_2_ showed the significant (*P* < 0.05) response surface models with high *R* ^2^ value of 0.9689 and 0.8838, respectively. Quadratic model were found to be maximum in adjusted *R* ^2^ and predicted *R* ^2^. However, Cubic model was found to be aliased. Therefore, quadratic model was chosen for further analysis.  Table 4 shows the adequacy of the model for VSS and COD reduction in ozonation process. The model Prob > *F* is less than 0.05, and lack of fit was calculated from the experimental error (pure error) and residual. The lack of fit of (LOF) *F*-values of these two models implies the variation of data around the fitted model and was significant. 

To test the estimated regression equation for the goodness of fit, Fisher's *F*-test was employed, and the multiple correlation coefficient *R* ^2^ was calculated. The model *F*-value of 34.56 and 8.45 implies that the model is significant for VSS and COD, respectively. 

### 3.2. Effect of pH on VSS and COD Reduction

**Figure 1:**
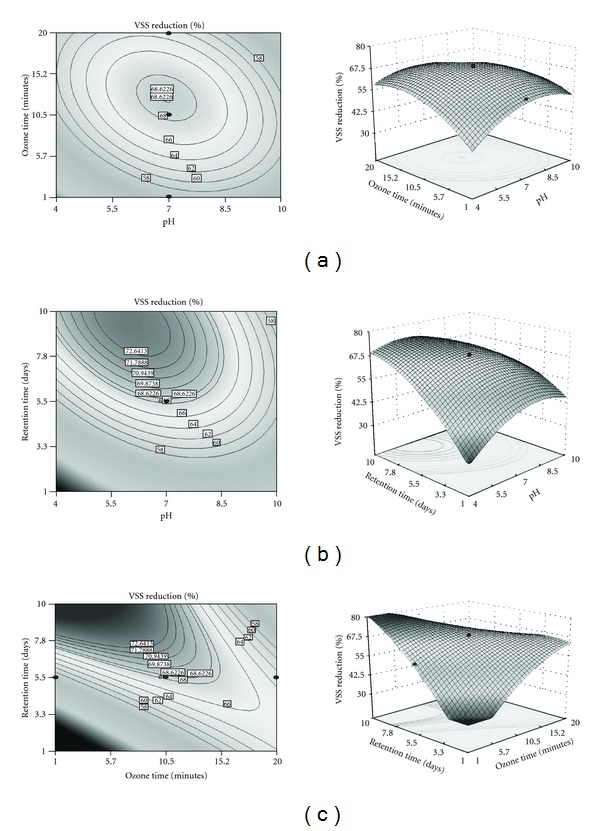
Contour and three-dimensional surface plot of VSS reduction. (a) Effect of pH and ozone time at fixed retention time (5.5 days). (b) Effect of pH and retention time at fixed ozone time (10.5 minutes). (c) Effect of ozone time and retention time at fixed pH 7.

**Figure 2:**
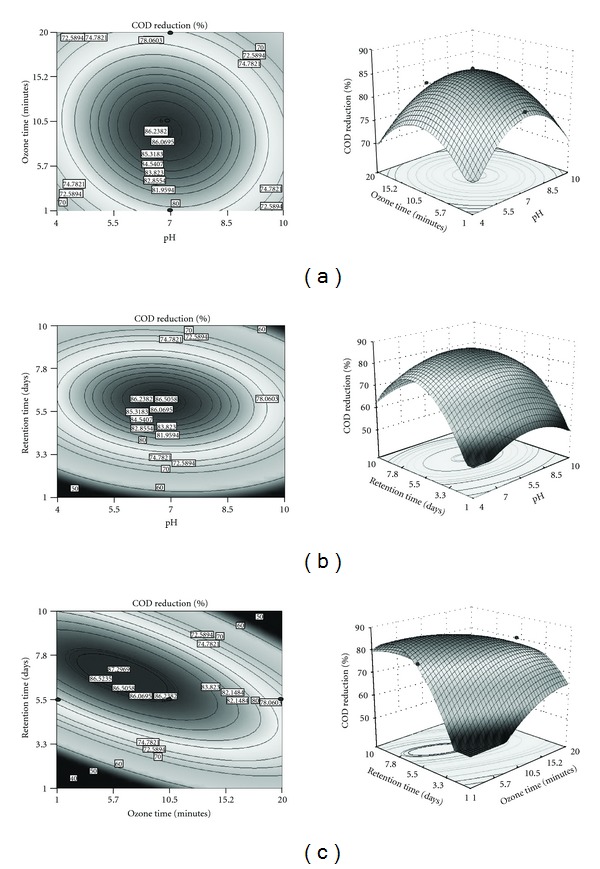
Contour and three-dimensional surface plot of COD reduction. (a) Effect of pH and ozone time at fixed retention time (5.5 days). (b) Effect of pH and retention time at fixed ozone time (10.5 minutes). (c) Effect of ozone time and retention time at fixed pH 7.

Biomass is mostly organic material, and an increase in it can be measured by VSS. For better explanation of the independent variables and their interactive effects on the VSS and COD reduction, contour and 3D plots are represented in Figures 1 and 2. pH was selected as one of the variable, and varied from acidic to alkaline (4–10). It was found that VSS reduction (76%) was observed in pH 6.9. As pH increased to alkaline conditions, VSS reduction decreased to 42%. Maximum COD reduction (86%) was achieved at acidic pH (6.7). Compared to acidic pH 6.7, neutral and alkaline pH had less COD reductions of 79% and 72%, respectively. This result implicates that acidic pH is favourable for VSS and COD reduction. 

The solubility of ozone is readily affected by pH. The influence of pH is the result of the relationship between oxidation potential and decomposition behaviour of ozone. In acidic pH, the ozone is available as molecular ozone, and in alkaline pH it decomposes into secondary oxidants such as OH^∙^, HO_2_^∙^, HO_3_^∙^, and HO_4_^∙^. Among these, OH^∙^ is an important one and has the highest oxidation potential of 2.8 V. The oxidizing potential of ozone decreased from 2.08 V at acidic pH to 1.4 V in alkaline solutions [25]. This indicates that the ozone reaction decreases with increasing pH resulting in generation of secondary oxidants. Hausler et al. [26] studied the ozonation of synthetic wastewater by varying the pH of the wastewater from basic to acidic resulting in significant improvement of ozonation rates. Ozone treatment of a Vinasse wastewater produced higher COD removal under acidic pH rather than alkaline pH [27]. Ozone penetrates into the microorganism, increases the osmosis of cell membrane, damages the uniformity of cell wall and releases the intracellular substances into wastewater, alters the permeability of the cell membrane, and ultimately results in the leakage of cell contents and reduced sludge biomass [28, 29]. Ozone can react directly with a substrate and decompose, under favourable conditions. Lucas et al. [30] observed the reduction of COD under the action of ozone at the acidic pH (4) of the winery wastewater. 

### 3.3. Effect of Ozonation Time on VSS and COD Reduction

The ozonation time required for reduction of VSS and COD was considered for the analysis of the variables and varied from 1 to 20 minutes. The effect of ozonation time on VSS and COD reduction is shown in Figures 1 and 2. The maximum VSS reduction (81%) was obtained at 12 minutes. As ozonation time increased, VSS reduction decreased to 32%. The main reason for sludge reduction during ozonation might be due to the rupture of microbial cell wall and release of extracellular and intercellular matter [29, 31]. Maximum COD reduction (87%) was obtained at 8 minutes. As ozonation time increased, COD reduction decreased up to 72%. It indicates that low ozonation time was in favour of VSS and COD reduction. This result reveals that prolongation of the sludge ozonation process causes the ozone to gradually lose its ability to oxidize sludge solids and soluble organic molecules. After introduction of ozone to the effluent, nutrients released by cell lysis and cell debris may alter the effluent characteristics and soluble organics released from the disrupted cells [28]. The ozonation of sludge leads to a decrease in the percentage of volatile suspended solids (VSS). Similar results were obtained as VSS decreased from 78% in raw sludge to 73% in ozonated sludge in municipal wastewater [9]. Yeom et al. [32] reported that the ozonated sludge showed 2-3 times greater biodegradation compared to the raw sludge in both aerobic and anaerobic conditions for 5 days.

Ozone may first react with the soluble portion of the activated sludge and then attack the particulate fraction. With an increase in ozone time, more intracellular substances were released. The soluble portion has a screening effect on the particulate matter attacked by ozone, which results in little improvement in sludge solubilisation at higher ozone doses [33]. Furthermore, it was reported that during ozonation, radical scavengers such as lactic acid and SO_4_^2−^ released from the microbial cell into the soluble part, which might have inhibited the future indirect reaction of ozone [28]. Yasui and shibata [34] proposed and developed an activated sludge process coupled with ozonation for sludge reduction. Research by Kamiya and Hirotsuji [35] showed that excess sludge production was reduced by 50% per day at ozone dose of 0.01 g O_3_/g TSS in the aerobic tank. When the ozone dose was kept as high as 0.02 g O_3_/g TSS, no excess sludge was produced. 

### 3.4. Effect of Retention Time on VSS and COD Reduction

Retention time is required for the analysis of VSS and COD reduction during ozonation process and varied from 1 to 20 days. The effect of retention time on VSS and COD reduction is shown in Figures 1 and 2. Maximum VSS reduction (80.5%) is obtained on the 10th day, and maximum COD reduction (86.5%) was achieved on the 6th day. As the retention time increased, COD reduction, decreased to 70%. Retention time was highly significant for biomass and COD reduction, and the microorganisms in the activated sludge have not established resistance to ozone. Gurak et al. [36] reported that extended solid retention time decreases biosludge in oil refinery sludge. This finding explores the possibility of using ozone to reduce excess sludge production in activated sludge processes. 

According to Müller [12], municipal wastewater was used and 30 days retention time, ozone concentration was 50 mg O_3_ L^−1^, there was no excess sludge production, and activated sludge microorganisms in the ozonation reactor would be killed and oxidized to organic substances [35]. Those organic substances produced from the sludge ozonation can then be degraded in the subsequent biological treatment. 

## 4. Conclusion

The growth of the sago processing industries resulted in high water pollution, as it generates large amounts of wastewater with extremely high concentrations of organic pollutants. Ozonation process was successfully employed for reduction of excess sludge production. RSM model used for the optimization of the operating condition for maximizing the VSS and COD reduction in ozonation condition. ANOVA results showed that the coefficient determination value, (*R* ^2^) of VSS and COD reduction were 0.9689 and 0.8838, respectively. By applying RSM, the optimum values were calculated. Experimental findings were in close agreement with the model prediction. Maximum reduction (81%) of VSS was achieved at acidic pH 6.9, 12 minutes ozonation time, and retention time of 10 days. Maximum COD reduction was attained at acidic pH 6.7, 8 minutes of ozonation time and retention time of 6 days. Acidic pH has a greater influence for VSS and COD reduction. At neutral pH, the efficiency of ozone is low when compared with that of acidic and alkaline pH. Ozonation time and retention time influences maximum sludge reduction and COD reduction. It was concluded from this study that reduction of excess sludge production in sago processing waste water by using ozonation process is very effective, and the variables pH, ozonation time, and retention time highly influence sludge reduction. Hence, this study was a novel attempt for excess sludge reduction process using ozonation process with RSM model and has helped to identify the most significant operating factors and optimum levels with minimum effort and time. 

## Figures and Tables

**Table 1:** Physicochemical characteristics of sago effluent.

Parameters	Values
pH	4.8
Total suspended solids (TSSs)	2689
Volatile suspended solids (VSSs)	1576
Chemical oxygen demand (COD)	13040
Settleable solids (SSs)	200
Phosphate	36.5
Nitrate	46
Sulphate	70
Carbohydrate	7.9
Starch	0.567

Except pH and settleable solids all other parameters are in mg/L. Settleable solids mL/L.

**Table 2:** Level of factors and their values used for the experiment.

Variables Coded values	Actual values for the coded values				
	−1.682	−1	0	+1	+1.682
pH (*X*_1_)	4	5.2	7	8.8	10
Ozonation time (*X*_2_) (minutes)	1	4.9	10.5	16.1	20
Retention time (*X*_3_) (days)	1	2.8	5.5	8.2	10

**Table 3:** The design matrix and observed values of the central composite design.

Run no.	Variables in uncoded levels						
	pH *X*_1_	Ozonation time *X*_2_	Retention time *X*_3_	VSS reduction % (*Y*_1_)		COD reduction % (*Y*_2_)	
				*Y*_experimental value_	*Y*_predicted value_	*Y*_experimental value_	*Y*_predicted value_
				(%)	(%)	(%)	(%)
1	7	10.5	5.5	75.12	76.31	85.40	86.17
2	10	10.03	5.5	50.39	50.98	74.80	74.69
3	8.8	16.1	2.8	44.77	40.60	67.50	73.30
4	5.2	4.9	8.2	96.59	99.93	86.00	87.12
5	7	10.5	5.5	76.42	76.31	65.00	68.89
6	7	10.5	5.5	76.35	76.31	86.30	86.17
7	7	10.5	5.5	76.79	76.31	86.40	86.17
8	7	10.5	5.5	76.79	76.31	86.10	86.17
9	8.8	4.9	8.2	60.40	53.91	53.40	59.95
10	3.97	10.5	5.5	60.51	59.39	65.00	62.56
11	7	10.5	10.4	89.22	87.19	80.40	89.30
12	7	10.5	0.96	21.18	26.31	40.40	49.40
13	8.8	16.1	8.2	86.80	82.99	64.50	66.66
14	5.2	16.1	2.8	76.78	76.31	85.00	83.31
15	8.8	4.9	2.8	93.18	89.44	63.19	65.78
16	5.2	16.1	8.2	96.26	97.29	80.70	80.63
17	7	19.92	5.5	76.24	76.07	64.00	56.03
18	5.2	4.9	2.8	59.41	67.66	80.40	75.59
19	7	1.08	5.5	47.58	46.31	63.20	63.42
20	7	10.5	5.5	76.79	76.31	86.40	86.17

**Table 4:** Adequacy of the model resulted for VSS and COD reduction.

	Source	Sum of squares	DF	Mean square	*F* value	*P*-value Prob > *F*	Source	SD	*R* ^2^	Adjusted *R* ^2^	Predicted *R* ^2^	PRESS
	Sequential model sum of square						Model summary statistics					
VSS reduction %	Mean	1.083*E* + 005	1	1.083*E* + 005								
	Linear	6471.83	3	2157.28	46.82	<0.0001	Linear	6.79	0.8977	0.8786	0.8184	1309.45
	2FI	75.90	3	25.30	0.50	0.6904	2FI	7.13	0.9083	0.8659	0.7773	1605.12
	Quadratic	436.69	3	145.56	6.48	0.0103	Quadratic	4.74	0.9689	0.9408	0.7656	1689.90
	Cubic	172.73	4	43.18	5.00	0.0407	Cubic	2.94	0.9928	0.9772	−0.5167	10933.58
	Residual	51.83	6	8.64								

	Total	1.155*E* + 005	20	5777.21								

COD reduction %	Mean	1.143*E* + 005	1	1.143*E* + 005	0.44	0.7255						
	Linear	160.84	3	53.61			Linear	11.00	0.0767	−0.0964	−0.4815	3106.68
	2FI	416.17	3	138.72	1.19	0.3531	2FI	10.81	0.2752	−0.0594	−1.4699	5179.18
	Quadratic	1276.36	3	425.45	17.47	0.0003	Quadratic	4.94	0.8838	0.7793	0.0979	1891.75
	Cubic	158.29	4	39.57	2.78	0.1266	Cubic	3.77	0.9593	0.8712	−7.8953	18652.95
	Residual	85.27	6	14.21								

	Total	1.164*E* + 005	20	5820.21								
